# Moving into an urban drug scene among people who use drugs in Vancouver, Canada: Latent class growth analysis

**DOI:** 10.1371/journal.pone.0224993

**Published:** 2019-11-14

**Authors:** Kanna Hayashi, Lianping Ti, Huiru Dong, Brittany Bingham, Andrew Day, Ronald Joe, Rolando Barrios, Kora DeBeck, M-J Milloy, Thomas Kerr

**Affiliations:** 1 British Columbia Centre on Substance Use, Vancouver, BC, Canada; 2 Faculty of Health Sciences, Simon Fraser University, Burnaby, BC, Canada; 3 Department of Medicine, University of British Columbia, Vancouver, BC, Canada; 4 School of Population and Public Health, University of British Columbia, Vancouver, BC, Canada; 5 Vancouver Coastal Health Authority, Vancouver, BC, Canada; 6 School of Public Policy, Simon Fraser University, Vancouver, Canada; University of Victoria, CANADA

## Abstract

**Background:**

Urban drug scenes are characterized by high prevalence of illicit drug dealing and use, violence and poverty, much of which is driven by the criminalization of people who use illicit drugs (PWUD) and the associated stigma. Despite significant public health needs, little is understood about patterns of moving into urban drug scenes among PWUD. Therefore, we sought to identify trajectories of residential mobility (hereafter ‘mobility’) among PWUD into the Downtown Eastside (DTES), an urban neighbourhood with an open drug scene in Vancouver, Canada, as well as characterize distinct trajectory groups among PWUD.

**Methods:**

Data were derived from three prospective cohort studies of community-recruited PWUD in Vancouver between 2005 and 2016. We used latent class growth analysis (LCGA) to identify distinct patterns of moving into the DTES among participants residing outside of DTES at baseline. Multivariable multinomial logistic regression was used to determine baseline factors associated with each trajectory group.

**Results:**

In total, 906 eligible participants (30.9% females) provided 9,317 observations. The LCGA assigned four trajectories: consistently living outside of DTES (52.8%); early move into DTES (11.9%); gradual move into DTES (19.5%); and move in then out (15.8%). Younger PWUD, those of Indigenous ancestry, those who were homeless or living in a single-room occupancy hotel (SRO), and those injecting drugs daily were more likely to move in then out of DTES (all p<0.05). Living in an SRO, daily injection drug use, and recent incarceration were also positively associated with early mobility (all p<0.05).

**Conclusions:**

Nearly half of the participants moved into the DTES. Younger PWUD and Indigenous peoples appeared to have particularly high mobility, as did those with markers of social-structural vulnerability and high intensity drug use. These findings indicate a need to tailor existing social and health services within the DTES and expand affordable housing options outside the DTES.

## Introduction

Urban drug scenes, which are typically characterized by a high prevalence of illicit drug dealing and use, violence and poverty, can be found in many urban cities around the world [1–3]. While the visibility and size may vary across settings, such drug scenes are conceptualized as distinct risk environments that can increase the vulnerability to poor health among the inhabitants [4–6]. Repressive law enforcement is a commonly employed measure to address complex problems within drug scenes, although this approach has been shown to increase gun violence and homicide rates [7], and they typically fail to produce sustained changes in public order within drug scenes [8]. Conversely, it is also common that drug scenes are sites for a variety of assertive health and social services due to the concentration of marginalized populations, including people who use illicit drugs (PWUD), and the range of health challenges many contend with [1,3,9]. While some European cities have been successful in closing down their drug scenes via coordinated law enforcement and public health efforts, globally, many urban drug scenes remain active. This raises concerns that these settings may attract young PWUD and facilitate ongoing cycles of problematic substance use [1].

PWUD are known to have high residential mobility (hereafter ‘mobility’), which has been shown to be associated with poor health outcomes such as HIV infection [10,11]. Previous research has suggested that there are likely many competing “push-pull” factors that influence PWUD’s decision to move, including access to drugs, poverty, employment, safety, previous involvement in the criminal justice system, and enrolment in addiction treatment programs [10,12,13]. While these diverse factors have been explored primarily through qualitative and ethnographic research, few quantitative studies have attempted to identify and characterize common patterns of moving into urban drug scenes among PWUD. This knowledge gap makes it challenging to address potential vulnerabilities associated with mobility among PWUD when planning a health service system within and outside of the urban drug scenes.

The Downtown Eastside (DTES) neighborhood in the city of Vancouver, Canada is known as one of the largest urban open drug scenes in North America, with widespread illicit drug use, as well as a high prevalence of poverty, violence, mental health and infectious diseases [14–16]. Also prevalent in the DTES are single room occupancy hotels (SROs), which are typically marked by sub-optimal and unhygienic conditions and yet represent the primary housing option for low-income populations, especially those dependent on government assistance [17,18]. Further, like other urban drug scenes elsewhere, the DTES is also home to a concentration of a range of low-threshold health and social services, including homeless shelters, supportive housing, food banks, on-demand addiction treatment, and innovative harm reduction services [16]. In this context, using data from three long-running, large prospective cohort studies of PWUD in Vancouver, we sought to characterize longitudinal patterns of moving into the DTES over the past decade, and identify socio-demographic, behavioural and social-structural factors associated with mobility patterns among PWUD.

It is important to note that the present study does not assume that moving into the DTES automatically represents changes in health or socioeconomic status of individuals. However, given the public health challenges associated with this neighborhood as described above, the present study aimed to better understand PWUD’s mobility patterns into the urban drug scene in a large cohort of community-recruited PWUD and thereby inform the planning of health and social services provision within and outside of the neighborhood to improve the health of PWUD. This applied policy perspective focusing on the health is important considering that punitive law enforcement has historically been a dominant approach to address illicit drug use [19] and has typically aimed to remove PWUD from certain neighborhoods or public spaces [20]. This approach has largely neglected PWUD’s experiences of social and economic sufferings that may have driven their problematic drug use, and has likely served to compound high mobility among this population [8]. While some qualitative and ethnographic studies have explored how social, structural and geographical factors interact with individual drug use and shape PWUD’s mobility [12,13], these research findings have not been corroborated by larger quantitative studies, likely due to the difficulty obtaining longitudinal mobility data from PWUD. In this regard, our study is uniquely positioned to contribute rare quantitative evidence to advance theories regarding factors associated with mobility patterns among PWUD [10,12,13]. Also, given that many of the health services targeted for PWUD requires continuum of care (e.g., opioid agonist maintenance therapy), an empirical examination of mobility patterns among PWUD such as this is important to inform more effective provision of health and social services among this population, which has long taken a back seat behind the law enforcement-based policy approach to illicit drug use [19].

## Materials and methods

### Study setting and design

Data were derived from three ongoing prospective cohort studies of PWUD in Vancouver: the Vancouver Injection Drug Users Study (VIDUS), the AIDS Care Cohort to evaluate Exposure to Survival Services (ACCESS), and the At-Risk Youth Study (ARYS). VIDUS started in 1996, while ACCESS and ARYS started in 2005. As described in detail previously [21–24], VIDUS enrols HIV-seronegative adults (≥18 years of age) who injected illicit drugs in the month prior to enrolment. ACCESS enrols HIV-seropositive adults who used an illicit drug other than or in addition to cannabis in the month prior to enrolment. ARYS enrols street-involved youth aged 14 to 26 who used an illicit drug other than or in addition to cannabis in the month prior to enrolment. For the ARYS cohort that includes youth aged less than 18 years, the local research ethics board has approved the lack of parent or guardian consent when the potential participant is considered ‘emancipated minors’ and fully capable of providing informed consent. Although these cohorts do not employ random sampling, it could be said that VIDUS and ACCESS cohort participants represent adults who inject drugs in our study setting, including those both in and out of healthcare (e.g., addiction treatment), and who are typically engaged in intensive substance use and at risk of a range of drug-related harm. ARYS participants represent drug-using youth who are socially marginalized and also at risk of a range of drug-related harm [15].

The studies employ harmonized data collection and follow-up procedures to allow for pooled analyses. Specifically, the cohorts are administered identical questionnaires with equal follow-up frequency (i.e., every 6 months). At baseline and semi-annually thereafter, participants complete an interviewer-administered questionnaire, which elicits a range of information including demographic data, substance use, and healthcare access, and provide blood samples for HIV and hepatitis C virus (HCV) serology. Participants receive a $40 (CDN) honorarium for each visit. All three cohorts have received approvals from the University of British Columbia/Providence Health Care Research Ethics Board.

### Study sample

Eligibility criteria for the present study included: completing at least four study visits between September 1, 2005 and May 31, 2016, and reporting having lived outside of DTES in the past six months at the first study visit during the study period. Given the lack of literature suggesting common mobility trajectories among our study population, we did not make any *a-priori* assumptions what the patterns would look like. Instead, we decided to use the latent class growth analysis (LCGA) to identify latent trajectory groups. For this reason, the data was restricted to participants who completed at least four study visits so we could test for mobility patterns that may follow a polynomial function with a degree greater than one (e.g., linear, quadratic, cubic functions) [25].

### Measures

The primary outcome for identifying DTES mobility patterns with LCGA was self-reported residence in DTES in the past six months (yes *vs*. no). This dichotomous indicator was a repeated measure derived from multiple study visits for each individual over the entire study period. We also included some self-reported baseline demographic characteristics into the LCGA, including age (per 10 years increase), sex (male *vs*. female), and self-identified ethnicity (Indigenous *vs*. non-Indigenous [i.e., all others who did not self-identify as Indigenous]). The term ‘Indigenous’ will be used throughout this paper to collectively describe the Indigenous peoples of Canada, inclusive of those who identify as ‘Aboriginal’, or First Nations, Métis and Inuit. This term is used while also acknowledging the diversity of cultures, languages and traditions that exist among Indigenous Canadians. We focused on Indigenous ancestry as previous research has shown much higher rates of mobility among Indigenous peoples in Canada compared to their non-Indigenous counterparts [26–28]. We note that for the present study, the Aboriginal Health team of the Vancouver Coastal Health Authority (VCH) (represented by the co-author, BB) played a key role in conceptualizing the study questions, designing the study, interpreting the results, and writing up the manuscript in order to ensure that the study addressed relevant issues among local Indigenous communities and was respectful and sensitive to related cultural concerns.

After the LCGA was completed, we considered a range of explanatory variables that we hypothesized might predict mobility trajectory group memberships as identified from the LCGA. The selection of the variables was informed by previous research on mobility of PWUD [10,12,13], input from the VCH Aboriginal Health team and observational experiences of healthcare providers within and outside of the DTES (co-authors AD, RJ and RB). All explanatory variables were assessed at baseline, referred to the past six months, and were dichotomized as yes *vs*. no unless otherwise stated. Socioeconomic factors included: being in a stable relationship, defined as being married, common law or having a regular partner; employment, defined as having a regular job, temporary work or self-employed; being on welfare or income assistance; current housing status (SRO *vs*. homeless *vs*. other unstable housing [e.g., in residential treatment] *vs*. stable housing [i.e., apartment or house; the reference category]); sex work; drug dealing; and incarceration, defined as spending at least one night in a youth detention facility, municipal jail, provincial prison or federal penitentiary. Substance use patterns were categorized as at least daily *vs*. less than daily *vs*. no use, and included: heroin use; non-medical use of prescription opioids, defined as using a prescription opioid when it was not prescribed for the participant or the participant took only for the experience or feeling it caused; stimulant (i.e., cocaine, crack or crystal methamphetamine) use; injection drug use; and alcohol use. Health service engagement included: opioid agonist therapy (i.e., methadone or buprenorphine-naloxone); any other addiction treatment or services except for opioid agonist therapy (detoxification/withdrawal management service *vs*. other services (e.g., counselling, Narcotic Anonymous, etc.) *vs*. not used); and any community health or social service use (e.g., food bank, drop-in centre, street nurse, etc.). Health status and outcomes included: non-fatal overdose; HIV seropositivity; HCV seropositivity; and having ever been diagnosed with a mental health disorder. We also included the calendar year of study enrolment (per year increase) to account for potential cohort effects.

### Statistical analyses

First, we compared demographic characteristics of the analytic sample to those excluded from the analysis due to the insufficient number of completed study visits, using the Pearson’s Chi-squared test (for categorical variables) and Wilxocon Rank Sum test (for continuous variables).

We used LCGA to identify distinct groups of participants with different longitudinal trajectories of mobility [29]. The time scale was the time from study enrolment for each individual. As mentioned above, we allowed group membership probabilities to vary as a function of characteristics of individuals by including age, sex and ethnicity in the LCGA. To determine the shape of trajectory group, we examined linear, quadratic and cubic function for each group first. If the higher-order parameters were not significant, we excluded those higher-order parameters and refit the model. To determine the optimal number of trajectory groups, we started with a single cubic trajectory model, and added groups in a step-wise fashion until the Bayesian Information Criterion (BIC) showed no substantial decrease. Next, we made sure the group membership probability was at least 5% to ensure the meaningful characterization and plausibility of identified trajectories. Also, the cut-off value for the average posterior probability was set as at least 0.7 as previously suggested [25,30]. Finally, we inspected visualized trajectory plots to determine whether the trajectory groups represented meaningful strata.

In order to identify a set of explanatory variables associated with DTES mobility patterns we built a multivariable multinomial logistic regression model [31]. The outcome of interest is a categorical variable representing the mobility patterns identified previously with LCGA. We used an *a priori*-defined backward model selection procedure based on examination of Akaike Information Criterion (AIC) to fit a multivariable model [32]. In brief, we constructed a full model including all variables that were associated with the outcome at *p*<0.10 in bivariable analyses. After examining the AIC of the model, we removed the variable with the largest *p*-value and built a reduced model. We continued this iterative process until we reached the lowest AIC score.

In the study interview, we also asked participants if they visited DTES while living outside of DTES in the past six months. If they did visit DTES, we also asked them to provide reasons. These data were summarized as descriptive statistics in a sub-analysis.

All *p*-values were two-sided. All statistical analyses were performed using SAS version 9.4 (SAS Institute, USA).

## Results

### Participant characteristics

In total, 906 participants were eligible for the present study and provided 9,317 observations. The median number of semi-annual study follow-up per participant was 10 (quartile [Q] 1–3: 5–14). As shown in Table 1, 280 (30.9%) were female, 247 (27.3%) self-identified as Indigenous, and the median age was 30 (Q 1–3: 22–44) years. In total, 697 participants were excluded from the analysis because they did not complete at least four study visits. Compared to those included (n = 906), those excluded were younger (median: 23 [Q1-3: 20–26]; p<0.001) and less likely to self-identify as Indigenous (n = 134, 19.2%, p<0.001). There was no significant difference with regard to sex between the two samples (female, n = 219, 31.4%, p = 0.825).

**Table 1:** Sample characteristics and bivariable multinomial logistic regression analyses of baseline factors associated with mobility trajectory groups among people who use drugs in Vancouver, Canada (n = 906).

Characteristic	n (%)	Odds Ratio (95% CI)		
		Group 2: Gradual move to DTES	Group 3: Move in then out	Group 4: Early move to DTES
Age (median, IQR)	30 (22–44)	n/a	n/a	n/a
**Male**	626 (69.1%)	n/a	n/a	n/a
**Indigenous ethnicity**	247 (27.3%)	n/a	n/a	n/a
Stable relationship*	300 (33.1%)	0.81 (0.54–1.22)	0.80 (0.54–1.20)	0.77 (0.49–1.22)
Employment*	392 (43.3%)	1.16 (0.80–1.69)	0.98 (0.67–1.43)	0.53 (0.34–0.84)
Being on welfare or income assistance*	644 (71.1%)	0.62 (0.42–0.92)	0.80 (0.54–1.20)	1.94 (1.12–3.38)
**Current housing status**				
Homeless	273 (30.1%)	1.77 (1.17–2.67)	2.28 (1.48–3.51)	1.31 (0.79–2.17)
SRO	101 (11.2%)	1.19 (0.58–2.42)	3.76 (2.14–6.60)	3.01 (1.64–5.55)
Other unstable housing	41 (4.5%)	0.58 (0.17–1.98)	1.99 (0.85–4.68)	1.62 (0.63–4.17)
Stable housing	478 (52.8%)	reference	reference	reference
Sex work*	82 (9.1%)	1.08 (0.55–2.11)	1.64 (0.92–2.95)	1.07 (0.50–2.26)
Drug dealing*	306 (33.8%)	1.50 (1.02–2.22)	1.71 (1.17–2.51)	1.43 (0.92–2.22)
Incarceration*	138 (15.2%)	2.50 (1.54–4.08)	1.75 (1.04–2.95)	3.16 (1.89–5.30)
Heroin use*				
≥Daily use	117 (12.9%)	1.53 (0.85–2.77)	2.01 (1.16–3.49)	3.39 (1.93–5.96)
<Daily use	241 (26.6%)	1.34 (0.87–2.06)	1.41 (0.92–2.16)	1.60 (0.97–2.63)
Not used	542 (59.8%)	reference	reference	reference
Non-medical use of prescription opioids*				
≥Daily use	42 (4.6%)	0.84 (0.28–2.53)	1.55 (0.66–3.64)	3.47 (1.59–7.61)
<Daily use	146 (16.1%)	1.37 (0.84–2.22)	0.84 (0.48–1.46)	1.67 (0.98–2.86)
Not used	709 (78.3%)	reference	reference	reference
Stimulant use*				
≥Daily use	224 (24.7%)	1.08 (0.61–1.91)	1.67 (0.90–3.09)	2.65 (1.31–5.35)
<Daily use	518 (57.2%)	1.00 (0.61–1.64)	1.59 (0.92–2.74)	1.64 (0.85–3.18)
Not used	163 (18.0%)	reference	reference	reference
Injection drug use*				
≥Daily use	147 (16.2%)	1.52 (0.87–2.64)	2.08 (1.25–3.46)	6.23 (3.39–11.46)
<Daily use	363 (40.1%)	0.91 (0.60–1.37)	0.79 (0.52–1.20)	2.44 (1.43–4.17)
Not used	394 (43.5%)	reference	reference	reference
Alcohol use*				
≥Daily use	97 (10.7%)	0.92 (0.48–1.76)	0.47 (0.21–1.06)	0.59 (0.28–1.25)
<Daily use	524 (57.8%)	0.93 (0.61–1.42)	1.10 (0.73–1.66)	0.64 (0.41–1.00)
Not used	283 (31.2%)	reference	reference	reference
OAT*	221 (24.4%)	0.68 (0.43–1.09)	0.72 (0.46–1.14)	1.35 (0.86–2.14)
Any other addiction treatment (excluding OAT)*				
Withdrawal management	34 (3.8%)	3.15 (1.34–7.40)	2.40 (0.97–5.95)	1.23 (0.34–4.41)
Other treatment/services	174 (19.2%)	1.13 (0.70–1.82)	1.08 (0.67–1.73)	1.20 (0.71–2.02)
Not used	692 (76.4%)	reference	reference	reference
Any community health or social service use*	774 (85.4%)	1.22 (0.71–2.09)	1.65 (0.92–2.95)	1.51 (0.79–2.87)
Non-fatal overdose*	79 (8.7%)	1.27 (0.69–2.35)	0.79 (0.39–1.62)	0.87 (0.40–1.90)
**HIV seropositivity**	231 (25.5%)	0.36 (0.21–0.62)	0.52 (0.32–0.83)	1.17 (0.74–1.83)
**HCV seropositivity**	443 (48.9%)	0.76 (0.52–1.12)	0.71 (0.48–1.03)	3.06 (1.91–4.89)
**Ever been diagnosed with a mental health disorder**	465 (51.3%)	0.68 (0.47–1.00)	0.71 (0.49–1.03)	0.80 (0.52–1.21)
**Calendar year of study enrolment**				
Median (IQR)	2006 (2006–2009)			
Per year later		1.11 (1.01–1.23)	1.07 (0.95–1.20)	1.08 (0.96–1.21)

DTES: Downtown Eastside. HIV: human immunodeficiency virus. HCV: hepatitis C virus. IQR: interquartile range. OAT: opioid agonist therapy. SRO: single room occupancy hotel.

* Denotes behaviours and events in the previous six months assessed at baseline

### Latent class growth analysis

**Fig 1:**
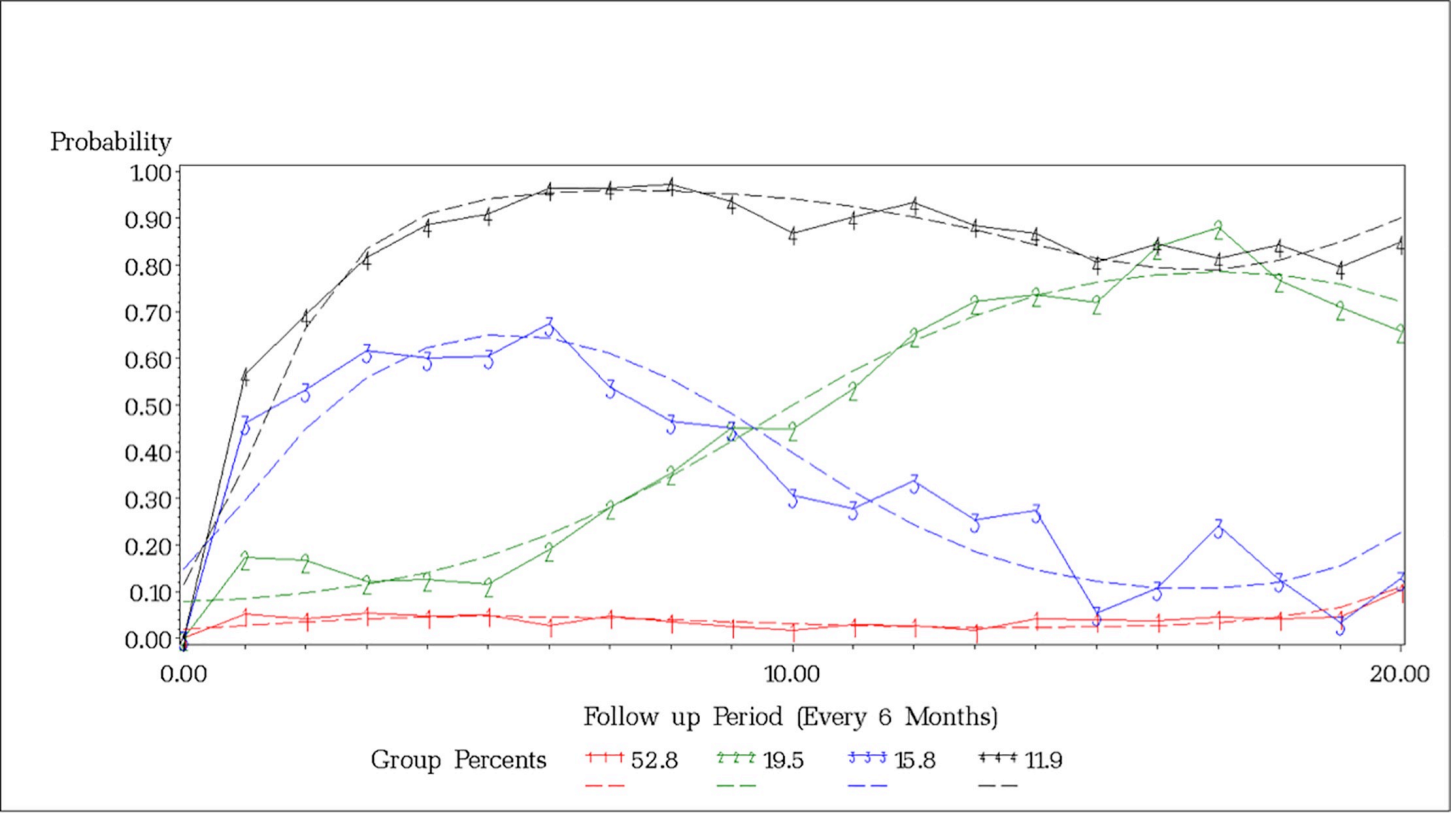
Four-group trajectories of moving into DTES, adjusting for baseline demographic characteristics (age, sex, and ethnicity). Red: Consistently living outside of DTES (Group 1). Green: Gradual move into DTES (Group 2). Blue: Move in then out (Group 3). Black: Early move into DTES (Group 4).

The LCGA resulted in a final cubic model with four trajectories (gradual move to DTES *vs*. move in then out *vs*. early move to DTES *vs*. consistently living outside DTES) (BIC = 3637.40; average posterior probability of ≥0.73). These four-group trajectories of mobility are visualized in Fig 1. As shown, the largest group (Group 1, 52.8%) represented those who had consistently lived outside of DTES. The second largest group (Group 2, 19.5%) included those who had gradually moved into DTES over time, with the slope of this trajectory increasing around 2.5 years after the baseline. Group 3 (15.8%) included individuals who had first moved into DTES but then moved out of DTES at a later time point. The smallest group (Group 4, 11.9%) included those who had made early move into DTES as represented by an early spike in the slope at around 6 months after the baseline.

In terms of the three baseline demographic covariates (age, sex and ethnicity) included in the LCGA model, age was significantly and negatively associated with membership in the “gradual move to DTES” group (odds ratio [OR]: 0.65; 95% confidence interval [CI]: 0.51–0.81) and the “move in then out” group (OR: 0.61; 95% CI: 0.48–0.79) compared to the “consistently living outside DTES” group. Indigenous ethnicity was significantly and positively associated with belonging to the “early move to DTES” group (OR: 2.38; 95% CI: 1.41–4.00) or the “move in then out” group (OR: 3.12; 95% CI: 1.75–5.57) compared to the “consistently living outside DTES” group.

### Multinomial logistic regression analysis

The results of bivariable and multivariable multinomial logistic regression analyses of baseline factors associated with mobility trajectory patterns (the reference category = the “consistently living outside DTES” group) are shown in Table 2. In multivariable analyses, compared to the “consistently living outside of DTES” group, factors independently associated with the “gradual move to DTES” group included: incarceration (adjusted odds ratio [AOR]: 2.20; 95% CI: 1.29–3.74) and HIV seropositivity (AOR: 0.49; 95% CI: 0.27–0.88). Factors independently associated with the “move in then out” group included: living in an SRO (AOR: 4.01; 95% CI: 2.19–7.37) or being homeless (AOR: 1.88; 95% CI: 1.17–3.03) as opposed to being stably housed, as well as daily injection drug use (AOR: 2.64; 95% CI: 1.45–4.81). Factors independently associated with the “early move to DTES” included: living in an SRO (AOR: 3.40; 95% CI: 1.73–6.68) as opposed to being stably housed, daily injection drug use (AOR: 3.80; 95% CI: 1.84–7.82), incarceration (AOR: 3.73; 95% CI: 2.08–6.70), and HCV seropositivity (AOR: 2.30; 95% CI: 1.24–4.27).

**Table 2:** Multivariable multinomial logistic regression analyses of baseline factors associated with mobility trajectory groups among people who use drugs in Vancouver, Canada (n = 906).

Characteristic	Adjusted Odds Ratio (95% CI)		
	Group 2: Gradual move to DTES	Group 3: Move in then out	Group 4: Early move to DTES
**Current housing status**			
Homeless	1.35 (0.84–2.17)	1.88 (1.17–3.03)	1.59 (0.87–2.89)
SRO	1.52 (0.73–3.17)	4.01 (2.19–7.37)	3.40 (1.73–6.68)
Other unstable housing	0.64 (0.18–2.27)	2.25 (0.92–5.53)	1.36 (0.46–4.03)
Stable housing	reference	reference	reference
Incarceration*	2.20 (1.29–3.74)	1.55 (0.88–2.72)	3.73 (2.08–6.70)
Injection drug use*			
≥Daily use	1.54 (0.80–2.97)	2.64 (1.45–4.81)	3.80 (1.84–7.82)
<Daily use	1.08 (0.66–1.78)	1.08 (0.66–1.77)	1.61 (0.85–3.04)
Not used	reference	reference	reference
**HIV seropositivity**	0.49 (0.27–0.88)	0.64 (0.37–1.11)	1.07 (0.62–1.86)
**HCV seropositivity**	0.91 (0.55–1.52)	0.78 (0.48–1.29)	2.30 (1.24–4.27)

DTES: Downtown Eastside. HIV: human immunodeficiency virus. HCV: hepatitis C virus. IQR: interquartile range. OAT: opioid agonist therapy. SRO: single room occupancy hotel.

* Denotes behaviours and events in the previous six months assessed at baseline

### Sub-analysis: Reasons for visiting DTES

There were a total of 7,695 reports regarding whether participants visited DTES while living outside of DTES in the past six months. Of these, 2,336 (30.4%) reported having not visited DTES, while 2,631 (34.2%) reported having visited DTES on a less than weekly basis, 1,648 (21.4%) reported visiting weekly or more frequently (but less than daily), and 990 (12.9%) reported visiting on a daily basis. Among those visiting DTES (n = 5,359), 4,981 identified one most relevant reason for visiting. The five most commonly reported reasons included: availability of drugs or alcohol (30.0%), family/partner/friend (15.9%), my doctor/clinic (10.9%), services (10.2%) and work/volunteering (6.6%). The descriptive statistics stratified by the factors that were independently associated with mobility patterns is presented in S1 and S2 Tables.

## Discussion

In our community-recruited sample of PWUD in Vancouver, nearly half of the participants who had resided outside the DTES at baseline moved into the DTES at some point over the 11-year study period. Among those who moved into the DTES, approximately one-third moved out thereafter. Both younger PWUD and Indigenous participants were more likely to have high mobility as they were more likely to move in and then out of the DTES. Overall, baseline factors that were independently and positively associated with moving into DTES included: unstable housing, recent incarceration, daily injection drug use and HCV seropositivity. In contrast, those living with HIV at baseline were less likely to move into the DTES.

To our knowledge, this is the first study that characterizes longitudinal patterns of mobility into an urban drug scene among a large sample of PWUD over an extended period of time. In particular, the four distinct mobility patterns that were identified by the LCGA are novel findings that we would have never been able to identify without using this analytical technique. The differences and similarities in the predictors of these mobility patterns provide some nuanced insight into how broader social-structural factors might influence mobility in this socially marginalized population and have some important implications for the planning of health and social services targeting this population.

While reasons for moving into DTES need to be further examined, the finding that the availability of drugs or alcohol, having family or friends in the neighborhood, as well as health and social services were among the most commonly cited reasons for visiting the DTES (though they were not the reasons for moving into the DTES) reflects the concentration of both illicit drug markets and the availability of low-threshold public services in this neighborhood [33,34]. Our findings suggest that these factors may have acted as pull factors in some PWUD’s decision to move into the DTES. For example, we found that markers of high intensity drug use, including daily injection drug use and HCV infection, were positively associated with early move to and the subsequent settlement in the DTES. For these PWUD, the proximity to illicit drug markets might have been a reason to relocate into the DTES, while the availability of some health and/or social services might have kept them in the neighborhood. Consistent with this interpretation, the escalating use of social services associated with moving into the DTES has also been found among individuals who were homeless and mentally ill [34]. However, it should be noted that only approximately 20% of participants reported coming to the DTES to access health and social services (10% for health services, and 10% for other services), with a greater number reporting that they came to the neighborhood for drugs/alcohol (30%) or to see family and friends (15.9%). In terms of healthcare, there is substantial evidence indicating that many PWUD experience discrimination in conventional healthcare settings [35,36]. It may be that the service providers in the DTES, given their extensive experience with the population, are providing more culturally appropriate and less discriminatory care, and by consequence many PWUD are actively seeking care there rather than relying on healthcare settings elsewhere.

Younger PWUD were more likely to gradually move into the DTES or move in then out of DTES. Our findings are in line with previous ethnographic studies describing that young PWUD in Vancouver symbolically associate the DTES neighborhood with danger and try to avoid visiting or seek to exit the neighborhood once having moved in [12,13]. This is in contrast to a commonly held claim that urban drug scenes attract youth [2]. In this regard, Sandberg and Pedersen criticized such claims as being based upon the almost hegemonic public representations of youth and PWUD as passive and irrational, and might serve to misinform public policies [20]. Our findings also indicate more nuanced mobility trajectories of younger PWUD, resisting the overly simplistic assumption that young PWUD are drawn to urban drug scenes. The findings call for more research into and policy action to address some potential social-structural drivers of young PWUD’s mobility into urban drug scenes, including homelessness associated with aging out of foster care [37].

Consistent with previous research [12,38], we observed large effects of baseline housing status on mobility trajectories. Specifically, PWUD who were homeless at baseline were more likely to move into the DTES but relocate out of the DTES later. This finding is congruent with a previous study showing persistently high residential mobility among homeless populations even after implementing housing interventions [39]. In our study setting, those who were homeless at baseline might have had to come to the DTES for shelters or other affordable housing options, such as SROs. Likewise, some of those who resided in SROs at baseline were also more likely to move in then out of the DTES. These findings may speak to the vulnerability of SRO tenants, particularly PWUD, to evictions and the subsequent residential instability [17,40]. The type of housing an individual is able to find in the DTES may influence settlement status in the DTES after the move. With an ongoing major housing crisis impacting our study setting, SROs are likely among the most common (or arguably the only) housing options available to our study population [18,41], which might have led to recurring housing instability. Even if they did not, poor conditions of many SROs have been shown to be associated with a range of negative health outcomes [17]. While future research should investigate the types and quality of housing that those participants typically resided in after moving within and outside of the DTES (and if they became or continued to be homeless after the move), it is likely that the housing quality was poor in the context of the ongoing housing crisis in our study setting. Therefore, our findings point to the need to establish affordable housing options that meet a minimum housing and health standards both within and outside of the DTES and make sure that a variety of housing types be offered to accommodate various needs (e.g., housing for families vs. singles, supportive housing with on-site supports and connections to health care services, etc.).

Similarly, we found that PWUD who had recently been incarcerated at baseline were more likely to move to and stay in the DTES, and the effect of incarceration was independent of housing status at baseline. Previous research has demonstrated that incarceration is strongly associated with persistent housing instability among people who were homeless or vulnerably housed at baseline [42]. Incarceration has also been shown to be associated with profound socioeconomic marginalization more broadly resulting from the criminal justice system involvement [43]. It is likely that PWUD who had been incarcerated had to resort to the DTES not only for transitional housing options but also for other social services for individuals released from custody, which are concentrated in the neighborhood. They might stay in the DTES due to the continued socioeconomic instability including difficulty obtaining employment [43].

We also found that Indigenous PWUD were more likely to move into the DTES early or move in then out of DTES. Previous research has suggested that Indigenous peoples in Canada generally experience higher rates of relocation within urban cities compared to their non-Indigenous counterparts, and their residential mobility is often involuntary, reflective of poor living conditions on reserves, as well as intergenerational in nature and influenced by colonial practices [26–28]. For example, Belanger, Awosoga and Head estimated the prevalence of homelessness among urban Indigenous peoples in Canada at 6.97%, while a national average was only 0.78% [28]. The overrepresentation of Indigenous peoples among the homeless has been attributed to the history of colonial policies, which have marginalized, displaced and dispossessed Indigenous peoples of their lands and thereby dismantled their family and cultural structures [28]. While further study is warranted to examine the extent to which socio-historic structures influence trajectories of Indigenous PWUD in our study population, it is noteworthy that previous studies associate frequent mobility of Indigenous peoples with poverty, racism, housing evictions, violence and criminal justice system involvement, as well as poor health outcomes [27,44]. Past research has also shown that Indigenous peoples often move back and forth between their home communities and inner-city centres [45,46]. Clearly, more research, both quantitative and qualitative, is needed to examine the unique contexts of moving into DTES and the consequences among Indigenous PWUD, accounting for broader socio-historic and structural factors. Also, given that the negative effects of colonial legacies extend across multiple generations, it is important to ensure that a reconciliation process, which has been called for by the Truth and Reconciliation Commission of Canada [47], be implemented.

Finally, those living with HIV at baseline were less likely to move into the DTES. Over the past decade, extensive multidisciplinary HIV care programs have been implemented in this setting following the emergence of a major epidemic of HIV infection with an aim to improve linkage, engagement and retention in evidence-based healthcare among those living with HIV [48]. Multidisciplinary teams provide outreach, peer support, clinical care, facilitation for housing and other social services, and addiction treatment services for PWUD [48]. Past research has shown high rates of engagement in HIV care among PWUD in this setting during a period of these community-wide HIV care initiatives [49]. Such integrated care models may have established social support networks outside of the DTES and prevented PWUD living with HIV from moving into the urban drug scene.

This study has some limitations. First, self-reported data may be influenced by some reporting bias, although such data have been shown to be mostly valid in studies involving PWUD [50]. Second, a non-random sample used in the present study limits the generalizability of our findings, although we note that it is virtually impossible to conduct random sampling of PWUD in the absence of registries of PWUD. In addition, our sample restrictions (i.e., completing at least four study visits) may have resulted in causing some selection bias. Given that the excluded were younger but less likely to self-identify as Indigenous, our findings may reflect the mobility patterns of older and/or Indigenous PWUD rather than younger and/or non-Indigenous participants, although it is difficult to estimate the direction of bias. Third, as with all observational research, the relationships between the explanatory variables and outcome assessed may be under the influence of unmeasured confounding, although we sought to address this bias with multivariable adjustment involving key predictors of mobility patterns. For example, we did not have data on histories of mobility prior to study enrolment, which may have influenced the observed associations. Also, while we used the baseline characteristics for the purpose of predicting distinct mobility trajectories, future research may examine time-varying effects of these characteristics. Further, because we did not have data on moving into areas other than the DTES, we were unable to identify the factors that were uniquely associated with moving into the DTES vs. other locations.

In sum, nearly half of PWUD in our sample moved into the DTES over the 11-year study period. Younger PWUD and Indigenous participants appeared to have particularly high rates of mobility, as did those with markers of social-structural vulnerability and high intensity drug use. These findings indicate a need to tailor the existing social and health services within the DTES for those moving in there and expand affordable housing options outside the DTES.

## Supporting information

S1 Table (XLSX)

S2 Table (XLSX)
